# Associations of physical activity and sedentary behavior during pregnancy with gestational diabetes mellitus among Asian women in Singapore

**DOI:** 10.1186/s12884-017-1537-8

**Published:** 2017-10-18

**Authors:** Natarajan Padmapriya, Jonathan Y. Bernard, Shen Liang, See Ling Loy, Shirong Cai, Iris Shen Zhe, Kenneth Kwek, Keith M. Godfrey, Peter D. Gluckman, Seang Mei Saw, Yap-Seng Chong, Jerry Kok Yen Chan, Falk Müller-Riemenschneider, on behalf of the GUSTO Study Group, Allan Sheppard, Amutha Chinnadurai, Anne Eng Neo Goh, Anne Rifkin-Graboi, Anqi Qiu, Arijit Biswas, Bee Wah Lee, Birit F. P. Broekman, Boon Long Quah, Borys Shuter, Chai Kiat Chng, Cheryl Ngo, Choon Looi Bong, Christiani Jeyakumar Henry, Cornelia Yin Ing Chee, Yam Thiam Daniel Goh, Doris Fok, Fabian Yap, George Seow Heong Yeo, Helen Chen, Hugo P. S. van Bever, Iliana Magiati, Inez Bik Yun Wong, Ivy Yee-Man Lau, Jeevesh Kapur, Jenny L. Richmond, Joanna D. Holbrook, Joshua J. Gooley, Kok Hian Tan, Krishnamoorthy Niduvaje, Leher Singh, Lin Lin Su, Lourdes Mary Daniel, Lynette Pei-Chi Shek, Marielle V. Fortier, Mark Hanson, Mary Foong-Fong Chong, Mary Rauff, Mei Chien Chua, Michael Meaney, Mya Thway Tint, Neerja Karnani, Ngee Lek, Oon Hoe Teoh, P. C. Wong, Pratibha Agarwal, Rob M. van Dam, Salome A. Rebello, Shang Chee Chong, Shu-E Soh, Sok Bee Lim, Chin-Ying Stephen Hsu, Victor Samuel Rajadurai, Walter Stunkel, Wee Meng Han, Wei Wei Pang, Yin Bun Cheung, Yiong Huak Chan, Yung Seng Lee

**Affiliations:** 1 Department of Obstetrics & Gynaecology, Yong Loo Lin School of Medicine, National University of Singapore, Singapore, Singapore; 2 Singapore Institute for Clinical Sciences, Agency for Science and Technology Research (A*STAR), Singapore, Singapore; 3 Biostatistics Unit, Yong Loo Lin School of Medicine, National University of Singapore, Singapore, Singapore; 4 KK Women’s and Children’s Hospital, Singapore, Singapore; 5 Duke-National University of Singapore, Singapore, Singapore; 6 Medical Research Council Lifecourse Epidemiology Unit, Southampton, UK; 7 NIHR Southampton Biomedical Research Centre, University of Southampton, Southampton, UK; 8 Liggins Institute, University of Auckland, Auckland, New Zealand; 9 Saw Swee Hock School of Public Health, National University of Singapore, Singapore, Singapore; 10 Institute for Social Medicine, Epidemiology and Health Economics, Charite University Medical Centre, Berlin, Germany

**Keywords:** Physical activity, Sedentary behavior, Gestational diabetes mellitus, Maternal glucose levels, Pregnancy

## Abstract

**Background:**

Few studies have investigated physical activity (PA) and sedentary behavior (SB) in relation to fasting (FG) and 2-h postprandial plasma glucose (2hPG) levels and gestational diabetes mellitus (GDM); we investigated these associations among Asian pregnant women.

**Methods:**

As part of the Growing Up in Singapore Towards healthy Outcomes cohort study, PA and SB (sitting and television times) were assessed by interviewer-administered questionnaire. During 75 g oral glucose tolerance tests at 26–28 weeks’ gestation we measured FG, 2hPG levels and GDM (FG ≥ 7.0 mmol/L and/or 2hPG ≥ 7.8 mmol/L). Associations were analysed by multiple linear and logistic regression.

**Results:**

Among the 1083 women studied, 18.6% had GDM. SB was not associated with FG, 2hPG and GDM. Higher categories of PA were associated with lower 2hPG and a lower likelihood of GDM (p-trend < 0.05), but not with FG levels. Compared to insufficiently active women, highly active women had lower 2hPG levels [β (95% CI): -0.32 (−0.59, −0.05), *p* = 0.020) and were less likely to have GDM [OR: 0.56 (0.32–0.98), *p* = 0.040]. Stratified analysis revealed no associations among under/normal-weight women, but significant associations among overweight/obese women; in those with BMI ≥23 kg/m^2^, sufficiently active and highly active women were less likely to have GDM [OR: 0.52, (0.29–0.93), *p* = 0.028, and OR: 0.34, (0.15–0.77), *p* = 0.010, respectively].

**Conclusion:**

Higher PA was associated with lower 2hPG levels and a lower prevalence of GDM, particularly in overweight/obese women. Further studies are warranted to confirm these findings, and to examine the effectiveness of PA promotion strategies for the prevention of gestational hyperglycemia.

## Background

Gestational diabetes mellitus (GDM) is defined by the onset or first detection of any degree of glucose intolerance during pregnancy [1]. It has been reported that almost 17.8% of all pregnancies are affected by GDM [2]; this rate ranges from 1 to 25.5% depending on the population studied and the diagnostic tests performed [2, 3]. GDM is associated with a higher risk of complications for both mother and offspring, including caesarean section, preeclampsia, perinatal morbidity, including macrosomia, neonatal hypoglycemia and jaundice [4], and developing type 2 diabetes later in life for mothers and offspring [5]. This emphasises the importance of research on modifiable factors to prevent GDM.

Physical activity (PA) is one of the modifiable lifestyle factors known to have direct and indirect impacts on insulin sensitivity and glucose homeostasis [6–8]. At least 150 min of moderate intensity PA per week is recommended for healthy pregnant women [9, 10], in line with the World Health Organization (WHO) recommendation for health benefit in general adult populations [11, 12]. Evidence suggests that regular PA during pregnancy may be inversely associated with the risk of GDM [6, 13–20]. The literature also highlights the role of pre-pregnancy body mass index (BMI) as a strong predictor of GDM [21, 22]. However, to our knowledge few studies have examined the association between PA and GDM according to pre-pregnancy BMI and their results were inconsistent [23–25].

Engaging in prolonged sedentary behavior (SB) may have an effect on metabolic health regardless of PA [26]. SB is defined as any waking behavior in a sitting or reclining posture with less than or equal to 1.5 metabolic equivalent tasks (METs) of energy expenditure [27]. A recent meta-analysis reported that prolonged sedentary time is independently associated with deleterious health outcomes, including type 2 diabetes [28]. However, the influence of sedentary behavior (SB) during pregnancy on GDM is poorly understood and the results of existing studies are inconsistent [24, 29, 30].

The majority of previous studies on PA and SB have been conducted in Western populations [17, 23–25, 29, 30], and only a few studies have been conducted among Asian women [31, 32]. However, Asian populations appear to be at a higher risk of developing GDM [21, 33–35], highlighting the public health relevance of examining the association of PA and SB with GDM in Asian women. Moreover, previous studies have investigated the association of PA and SB with gestational glycaemia mainly based on an established glycemic level cut-off points for GDM diagnosis [36]. However, the international multicenter Hyperglycemia and Adverse Pregnancy Outcomes (HAPO) study reported that maternal fasting and stimulated glucose levels even below previous GDM cut-off points were linearly associated with increased adverse pregnancy and neonatal outcomes [37]. This suggests the need to additionally investigate continuous maternal glucose levels regardless of GDM cut-off points.

Based on the above evidence and gaps in the existing literature, we examined the associations of PA and SB during pregnancy with maternal glycemic levels and GDM among Asian women. Additionally, we investigated the magnitude of these associations according to pre-pregnancy BMI subgroups.

## Methods

### Study design and population

The Growing Up in Singapore Towards healthy Outcomes (GUSTO) mother-offspring cohort study recruited pregnant women attending their first-trimester antenatal dating ultrasound scan clinics at two major public maternity units in Singapore, KK Women’s and Children’s Hospital (KKH) and National University Hospital (NUH) from June 2009 to September 2010. Pregnant women aged 18 years and above, major ethnic groups (Chinese, Malay and Indian), Singapore citizen or permanent residents who had the intention of delivering in KKH/NUH and staying in Singapore for at least next 5 years, and who had agreed to donate their birth tissues were invited to participate in the study. Women who had type I diabetes mellitus, or who were receiving chemotherapy or psychotropic drugs were excluded. The GUSTO study was designed to investigate the early determinants of child health and development. More details on the study are available elsewhere [38, 39]. The study protocol was approved by the ethics committees of the hospitals involved: SingHealth Centralized Institutional Review Board and the National Healthcare Group Domain Specific Review Board in Singapore. All participants gave written informed consent.

### Data collection

As part of interviewer-administered questionnaires at recruitment, women were asked about their age, ethnicity, educational level, pre-pregnancy weight, family history of diabetes, and maternal history of GDM in previous pregnancies. When seen at 26–28 weeks gestation, women were asked questions about cigarette smoking during pregnancy and whether they were exposed to cigarette smoking at home or in their workplace during pregnancy; answers were combined to determine active/passive cigarette smoking during pregnancy.

Participants’ dietary energy intake was ascertained based on a 24-h dietary recall; this was administered by trained interviewers at a face-to-face interview at 26–28 weeks of gestation. Various portion sizes of standardized household measuring utensils and food pictures were used to assist women in quantifying their food and beverage intake. Nutrient analysis software (Dietplan, version 7, Forestfield Software) with a food composition database of locally available foods was used to calculate total daily energy intake. Additionally, for food items not found in the database, nutrient information was obtained from food labels or the USDA national nutrient database [19].

Parity data was collected from hospital medical records. Height (to the nearest 0.1 cm) and weight (to 0.01 kg) were measured by trained research staff using a stadiometer (SECA model 213, Hamburg, Germany) and a weighing scale (SECA model 803), respectively. Self-reported pre-pregnancy weight, and height measured at the 26–28 weeks gestational visit were used to calculate pre-pregnancy BMI (kg/m^2^). BMI was categorized as underweight, normal weight, overweight and obese (<18.5, 18.5–23, 23–27.5 and ≥27.5, respectively) according to Asian cut-offs [40–42]. Gestational weight gain until 26–28 weeks was calculated by subtracting self-reported pre-pregnancy weight from the weight measured at 26–28 weeks’ gestation [43].

### Assessment of maternal blood glucose concentrations

Participants underwent a 75-g oral glucose tolerance testing (OGTT) at 26–28 weeks’ gestation; overnight fasting (8–10 h) and 2-h postprandial blood specimens were collected. Colorimetry [Advia 2400 Chemistry system (Siemens Medical Solutions Diagnostics) and Beckman LX20 Pro analyzer (Beckman Coulter)] were used to measure both fasting glucose (FG) and 2-h postprandial glucose (2hPG) concentrations. Glucose concentrations were used to classify GDM according to the 1999 WHO standard criteria: ≥7.0 mmol/L for FG and/or ≥7.8 mmol/L for 2hPG [36, 44].

### Assessment of physical activity and sedentary behavior

PA questions were part of a structured questionnaire administered by trained interviewers at 26–28 weeks’ gestation; the questionnaire included questions on physical activities during the past 6 months of pregnancy. The detailed measurement of PA and SB in the cohort has been reported elsewhere [45]. Briefly, total physical activities were categorized as light-moderate (leaves the person tired but not exhausted, e.g. walking, gardening and golf), moderate (leaves the person exhausted but not breathless, e.g. brisk walking, easy swimming and dancing) and vigorous intensity (makes the heart beat rapidly and leaves the person breathless, e.g. jogging, vigorous swimming, cycling and aerobics). The participants reported the frequency and duration of the three different intensity levels of all physical activities. Frequency was categorized in the questionnaire as never, once every 2–3 months, once a month, once a fortnight, 1–2 times per week, 3–6 times per week, once a day, and more than once a day. Based on these categories an average frequency per week was converted into 0, 0.1, 0.25, 0.5, 1.5, 4.5, 7, and 10.5 times per week, respectively. Women reported an average duration of each period of activity, and the answers were standardized to the nearest 0.5 h. Total hours spent in each intensity level of PA per week was derived by multiplying frequency per week and duration per episode.

An absolute metabolic equivalent task (MET) value was assigned for light-moderate, moderate and vigorous intensity activities (3.3, 4.0 and 8.0, respectively), adapted from the protocol for the International Physical Activity Questionnaire (IPAQ) short form, with 1.0 MET corresponding to resting energy expenditure [46]. Energy expended on each level of PA intensity in MET-hours per week was calculated by multiplying total hours spent on specific intensity per week with its corresponding MET value. Energy expended per week in all three PA intensity levels were summed to estimate total energy expenditure (TEE) on PA per week (light-moderate + moderate + vigorous MET-hours/week), which was then converted into MET-minutes per week. TEE on PA was categorized into insufficiently active (<600 MET-minutes/week), sufficiently active (600 to <3000 MET-minutes/week), and highly active (≥3000 MET-minutes/week). This approach is based on the WHO PA recommendation of a minimum of 600 MET-minutes per week for health benefits in adults [11, 12], and on the IPAQ definition of at least 3000 MET-minutes per week for highly active adults [46]. For example, women who engaged in 30 min of moderate intensity PA at least for 5 days per week were classified as sufficiently active, and women who additionally engaged in 120 min of light-moderate intensity PA daily were classified as highly active.

Sedentary behavior was determined by asking participants to report total sitting time per day during the past 6 months of pregnancy during an interview at 26–28 weeks of gestation; the answers were standardized to the nearest 0.5 h. A separate question determined time spent watching television per day; answers were categorized as none, less than an hour, 1–2 h, 2–3 h, 3–4 h, 4–5 h, and more than 5 h. Based on these categories, an average television viewing duration was converted into 0, 0.5, 1.5, 2.5, 3.5, 4.5 and 5.5 h per day, respectively. In the absence of cut-offs for SB, daily total sitting time during pregnancy was categorized into tertiles to define low, medium and high total sitting time (<7, 7–10, ≥10 h/day, respectively). Higher television viewing time per day during pregnancy was determined based on an upper tertile, which was ≥3 h per day in this sample. This category corresponds with the existing literature, in which 20 or more hours of television viewing time per week during pre-pregnancy has been associated with GDM [47].

### Statistical analysis

Frequencies and percentages were calculated for categorical variables, and means and standard deviations for continuous variables. Differences between women with and without GDM were compared using an independent t-test for continuous variables and chi-square tests for categorical variables. Multivariable linear regression was used to investigate the associations of PA, total sitting time and television time with FG and 2hPG concentrations. Multivariable logistic regression was used to assess the associations of PA, total sitting time and television time with GDM. Based on the literature, models were adjusted for following confounders: maternal age, ethnicity, education, pre-pregnancy BMI (continuous), parity at recruitment, history of GDM in previous pregnancy, family history of diabetes, dietary energy intake, exposure to active/passive smoking during pregnancy and pregnancy weight gain at 26–28 gestational visit [24, 48–50]. Additionally, PA and total sitting time, and PA and television viewing time were mutually adjusted in separate models, to explore the independent associations of PA, total sitting time, and television viewing time with all outcome measures. The magnitude of these associations according to pre-pregnancy BMI subgroups was tested by stratifying regressions according to BMI subgroups, though the interaction between PA/SB and BMI subgroups on FG/2hPG/GDM was not statistically significant (*p* > 0.05). BMI subgroups were combined into two groups, under/normal weight and overweight/obese to enhance statistical power. Based on the observed standard deviations, a targeted statistical power of ≥80% and a type I error of 5%, our study is powered to detect differences of 0.05 and 0.14 mmol/L for fasting and 2-h glucose, respectively. For a subgroup of 100 participants, the differences that could be detected were 0.14 and 0.43 mmol/L, respectively. All association tests were two-tailed, and confidence intervals were calculated at the 95% level. All statistical analyses were performed using Statistical Package for the Social Sciences v19 (IBM, Chicago, IL, USA).

## Results

**Fig. 1:**
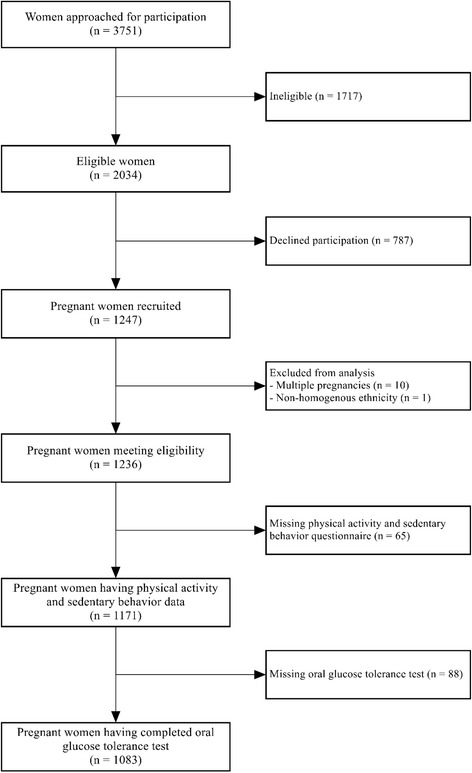
Study flow diagram

There were 1236 women eligible for the study; among them, 1083 (87.6%) participants completed the OGTT and answered PA and SB questions and were thus included in the present analysis (Fig. 1). Excluded women were similar to included subjects in relation to their age, education, parity, BMI, history of GDM, family history of diabetes and dietary energy intake (p > 0.05), but they were more likely to be of Malay or Indian ethnicity (*p* = 0.033). In our sample 18.6% (*n* = 201) of the women had been diagnosed with GDM; these women were more likely to be older, of Indian ethnicity, highly educated, to have a higher BMI and a history of GDM and to not report active/passive smoking during pregnancy (*p* < 0.05) (Table 1).

**Table 1:** Characteristics of pregnant women according to GDM in the GUSTO cohort study (*n* = 1083)

	GDM absent *n* = 882 (81.4%)	GDM present *n* = 201 (18.6%)	*p*-value
Mean age in years (SD)	30.3 (5.1)	32.5 (4.7)	<0.001
Ethnicity (n, %)			0.001
Chinese	494 (56.0)	126 (62.7)	
Malay	245 (27.8)	31 (15.4)	
Indian	143 (16.2)	44 (21.9)	
Education (n, %)			0.008
No-formal/Primary/Secondary	279 (31.6)	46 (22.9)	
GCE ‘A’ levels/polytechnic/Diploma/Technical education	312 (35.4)	72 (35.8)	
University	278 (31.5)	83 (41.3)	
Missing data	13 (1.5)	0 (0.0)	
BMI in kg/m^2^ (n, %)			<0.001
< 18.5	105 (11.9)	16 (7.9)	
18.5–23	431 (48.9)	81 (40.3)	
23.0–27.5	179 (20.3)	54 (26.9)	
≥ 27.5	97 (11.0)	43 (21.4)	
Missing data	70 (7.9)	7 (3.5)	
Parity at recruitment (n, %)			0.128
0	407 (46.1)	85 (42.3)	
≥ 1	463 (52.5)	116 (57.7)	
Missing data	12 (1.4)	0 (0.0)	
History of GDM (n, %)			
No	834 (94.6)	181 (90.0)	<0.001
Yes	18 (2.0)	18 (9.0)	
Missing data	30 (3.4)	2 (1.0)	
Family history of diabetes (n, %)			
No	603 (68.4)	133 (66.2)	0.072
Yes	253 (28.7)	67 (33.3)	
Missing data	26 (2.9)	1 (0.5)	
Dietary energy intake in kcal (Mean, SD)	1881.3 (604.5)	1815.2 (520.7)	0.118
Missing data (n,%)	6 (0.7)	2 (1.0)	
Active/ passive smoking during pregnancy (n, %)			0.001
No	503 (57.0)	138 (68.6)	
Yes	350 (39.7)	52 (25.9)	
Missing data	29 (3.3)	11 (5.5)	
Pregnancy weight gain at week 26–28 (Mean, SD)	8.7 (4.5)	8.0 (4.4)	0.061
Missing (n,%)	79 (9.0)	7 (3.5)	
Physical activity during pregnancy (n, %)			0.125
Insufficiently active	289 (32.8)	78 (38.8)	
Sufficiently active	415 (47.1)	95 (47.3)	
Highly active	167 (18.9)	25 (12.4)	
Missing data	11 (1.2)	3 (1.5)	
Total sitting time during pregnancy (n, %)			0.193
Low (<7 h/day)	252 (28.6)	43 (21.4)	
Medium (7–10 h/day)	239 (27.1)	62 (30.8)	
High (≥10 h/day)	389 (44.1)	95 (47.3)	
Missing data	2 (0.2)	1 (0.5)	
Total television viewing time (n, %)			0.066
Low (<3 h/day)	593 (67.2)	142 (70.6)	
High (≥3 h/day)	289 (32.8)	58 (28.9)	
Missing data	0 (0.0)	1 (0.5)	

*GDM* gestational diabetes mellitus, *SD*, standard deviation, *GCE ‘A’ levels* General Certificate of Education-Advance levels, *BMI* body mass index

*p*-values were determined by Chi-square or independent t-test

### Associations of PA and SB during pregnancy with maternal blood glucose levels

PA and television time during pregnancy were not significantly associated with FG levels in the overall sample and in BMI subgroups (Table 2). Total sitting time was inversely associated with FG levels in the unadjusted model (overall *p* = 0.029; p for trend =0.035), however, after adjustment this was attenuated with no linear trend (overall *p* = 0.074; p for trend = 0.183). Associations with 2hPG are shown in Table 3. Higher PA during pregnancy was associated with lower 2hPG (p for trend = 0.016). Compared with insufficiently active women, highly active women had lower 2hPG levels [β (95% CI): -0.32 (−0.59, −0.05), *p* = 0.020]. However, a sufficient level of PA was not significantly associated with 2hPG levels [β: −0.16 (−0.37, 0.04), *p* = 0.116]. Stratified analysis revealed associations only among overweight/obese women, but not among underweight/normal weight women (β: −0.51 (−1.00, −0.01), *p* = 0.044, and −0.17 (−0.49, 0.15), *p* = 0.291, respectively).

**Table 2:** Associations of physical activity and sedentary behavior with fasting glucose levels (mmol/l) during pregnancy in the GUSTO study

Variables	Overall sample					Underweight/Normal weight			Overweight/obese			P for interaction
	n	Unadjusted		Adjusted^a^		Adjusted^b^			Adjusted^b^			
		β (95% CI)	*P*-value	β (95% CI)	*P*-value	n	β (95% CI)	*P*-value	n	β (95% CI)	*P*-value	
Physical activity			0.759		0.806			0.525			0.863	0.933
Insufficiently active	325	Reference		Reference		202	Reference		123	Reference		
Sufficiently active	436	0.01 (−0.06, 0.08)	0.822	0.01 (−0.06, 0.08)	0.754	284	0.03 (−0.03, 0.10)	0.321	152	−0.001 (−0.15, 0.15)	0.988	
Highly active	160	0.04 (−0.06, 0.13)	0.463	0.03 (−0.06, 0.12)	0.512	99	0.04 (−0.05, 0.13)	0.350	61	0.05 (−0.15, 0.25)	0.631	
p for trend			0.492		0.520			0.286			0.681	
Total sitting time			**0.029**		0.074			0.106			0.202	0.457
Low (<7 h/day)	251	Reference		Reference		153	Reference		98	Reference		
Medium (7–10 h/day)	265	0.02 (−0.06, 0.11)	0.627	0.04 (−0.04, 0.13)	0.304	160	0.06 (−0.01, 0.14)	0.111	105	0.002 (−0.17, 0.18)	0.980	
High (≥10 h/day)	417	−0.07 (−0.15, 0.003)	0.060	−0.04 (−0.12, 0.04)	0.285	277	−0.01 (−0.08, 0.06)	0.843	140	−0.13 (−0.30, 0.04)	0.144	
p for trend			**0.035**		0.183			0.598			0.119	
Television time			0.446		0.367			0.134			0.791	0.399
Low (<3 h/day)	640	Reference		Reference		411	Reference		229	Reference		
High (≥3 h/day)	294	0.03 (−0.04, 0.09)		0.03 (−0.04, 0.10)		180	0.05 (−0.02, 0.11)		114	−0.02 (−0.17, 0.13)		

*β* beta-coefficient, *CI* confidence interval, *BMI* body mass index

A^a^Adjusted for maternal age, ethnicity, education, pre-pregnancy BMI, parity at recruitment, history of GDM in previous pregnancy, family history of diabetes, dietary energy intake, active/passive smoking during pregnancy and pregnancy weight gain at 26–28 gestational visit

B^b^Adjusted for maternal age, ethnicity, education, parity at recruitment, history of GDM in previous pregnancy, family history of diabetes, dietary energy intake, active/passive smoking during pregnancy and pregnancy weight gain at 26–28 gestational visit

*p*-values were determined by multivariable linear regression

**Table 3:** Associations of physical activity and sedentary behavior with 2-h-postprandial glucose levels (mmol/l) during pregnancy in the GUSTO study

Variables	Overall sample					Underweight/Normal weight			Overweight/obese			P for interaction
	n	Unadjusted		Adjusted^a^		Adjusted^b^			Adjusted^b^			
		β (95% CI)	*P*-value	β (95% CI)	*P*-value	n	β (95% CI)	*P*-value	n	β (95% CI)	*P*-value	
Physical activity			**0.032**		0.056			0.570			0.109	0.607
Insufficiently active	325	Reference		Reference		202	Reference		123	Reference		
Sufficiently active	436	−0.13 (−0.35, 0.08)	0.222	−0.16 (−0.37, 0.04)	0.116	284	−0.07 (−0.31, 0.17)	0.576	152	−0.28 (−0.66, 0.10)	0.152	
Highly active	160	**−0.38 (−0.67, −0.10)**	**0.009**	**−0.32 (−0.59, −0.05)**	**0.020**	99	−0.17 (−0.49, 0.15)	0.291	61	**−0.51 (−1.00, −0.01)**	**0.044**	
p for trend			**0.010**		**0.016**			0.295			**0.036**	
Total sitting time			0.091		0.172			0.082			0.885	0.547
Low (<7 h/day)	251	Reference		Reference		153	Reference		98	Reference		
Medium (7–10 h/day)	265	0.25 (−0.01, 0.52)	0.056	0.23 (−0.02, 0.47)	0.073	160	**0.31 (0.02, 0.60)**	**0.037**	105	0.10 (−0.34, 0.55)	0.652	
High (≥10 h/day)	417	**0.24 (0.001, 0.48)**	**0.049**	0.17 (−0.06, 0.40)	0.137	277	0.25 (−0.01, 0.52)	0.062	140	0.02 (−0.41, 0.45)	0.931	
p for trend			0.069		0.189			0.099			0.971	
Television time			0.284		0.649			0.925			0.571	
Low (<3 h/day)	640	Reference		Reference		411	Reference		229	Reference		0.363
High (≥3 h/day)	294	−0.11 (−0.32, 0.10)		0.05 (−0.15, 0.25)		180	−0.01 (−0.25, 0.23)		114	0.11 (−0.26, 0.47)		

*β* beta-coefficient, *CI* confidence interval, *BMI* body mass index

A^a^Adjusted for maternal age, ethnicity, education, pre-pregnancy BMI, parity at recruitment, history of GDM in previous pregnancy, family history of diabetes, dietary energy intake, active/passive smoking during pregnancy and pregnancy weight gain at 26–28 gestational visit

b^b^Adjusted for maternal age, ethnicity, education, parity at recruitment, history of GDM in previous pregnancy, family history of diabetes, dietary energy intake, active/passive smoking during pregnancy and pregnancy weight gain at 26–28 gestational visit

*p*-values were determined by multivariable linear regression

Compared to women with low sitting time, women with high sitting time had significantly higher 2hPG levels; this association was no longer apparent after adjusting for potential confounders in the overall sample [unadjusted β 95% CI: 0.24 (0.001, 0.48), *p* = 0.049, adjusted β: 0.17 (−0.06, 0.40), *p* = 0.137; p for trend = 0.189]. Among under/normal weight women, those categorized as having medium and high sitting time had higher 2hPG as compared to women with low sitting time [adjusted β 95% CI: 0.31, (0.02, 0.60), *p* = 0.037, and 0.25 (−0.01, 0.52), *p* = 0.062, respectively]; however, there was no linear association (p for trend = 0.099). Sitting time was not associated with 2hPG among overweight/obese women. Television time during pregnancy was not associated with the 2hPG level.

### Associations of PA and SB with GDM

Table 4 shows that PA during pregnancy was inversely associated with the likelihood of having GDM (p for trend = 0.041): compared with insufficiently active women, highly active women were less likely to have GDM [adjusted OR (95% CI): 0.56 (0.32–0.98), *p* = 0.040]. However, a sufficient level of PA was not associated with GDM in the overall sample (OR: 0.82 (0.56–1.20), *p* = 303). Among overweight/obese women, sufficiently active and highly active women during pregnancy were less likely to have GDM as compared to insufficiently active women [adjusted OR: 0.52 (0.29–0.93), *p* = 0.028, and 0.34 (0.15–0.77), *p* = 0.010, respectively; p for trend = 0.004]. Among under/normal weight women on the other hand, PA during pregnancy was not associated with GDM [OR: 1.18 (0.71–1.98), *p* = 0.526, and 0.89 (0.42–1.90), *p* = 0.766, for sufficiently and highly active women, respectively]. Compared to women in the low sitting time group, women in the medium and high sitting groups had increased odds of having GDM, but this was not statistically significant [OR: 1.45 (0.90, 2.34), *p* = 131 and 1.42 (0.90, 2.22), p = 131, respectively]. There was no evidence for associations of television viewing time with GDM.

**Table 4:** Associations of physical activity and sedentary behavior during pregnancy with GDM in the GUSTO study

Variables	Overall sample					Underweight/Normal weight			Overweight/obese			P for interaction
	Cases n (%)	Unadjusted		Adjusted^a^		Adjusted^b^			Adjusted^b^			
		OR (95% CI)	*P*-value	OR (95% CI)	*P*-value	Cases n (%)	OR (95% CI)^a^	*P*-value	Cases n (%)	OR (95% CI)^a^	*P*-value	
Physical activity			0.107		0.118			0.673			**0.015**	0.098
Insufficiently active	71 (21.8)	1.0		1.0		30 (14.9)	1.0		41 (33.3)	1.0		
Sufficiently active	86 (19.7)	0.88 (0.62, 1.25)	0.475	0.82 (0.56, 1.20)	0.303	50 (17.6)	1.18 (0.71, 1.98)	0.526	36 (23.7)	**0.52 (0.29, 0.93)**	**0.028**	
Highly active	22 (13.8)	**0.57 (0.34, 0.96)**	**0.035**	**0.56 (0.32, 0.98)**	**0.040**	12 (12.1)	0.89 (0.42, 1.90)	0.766	10 (16.4)	**0.34 (0.15, 0.77)**	**0.010**	
p for trend			**0.045**		**0.041**			0.975			**0.004**	
Total sitting time			0.197		0.246			0.532			0.304	0.660
Low (<7 h/day)	39 (15.5)	1.0		1.0		19 (12.4)	1.0		20 (20.4)	1.0		
Medium (7–10 h/day)	55 (20.8)	1.42 (0.91, 2.24)	0.126	1.45 (0.90, 2.34)	0.131	24 (15.0)	1.29 (0.65, 2.56)	0.476	31 (29.5)	1.71 (0.86, 3.40)	0.123	
High (≥10 h/day)	87 (20.9)	1.43 (0.95, 2.17)	0.089	1.42 (0.90, 2.22)	0.131	49 (17.7)	1.42 (0.77, 2.63)	0.261	38 (27.1)	1.38 (0.71, 2.69)	0.341	
p for trend			0.114		0.172			0.271			0.438	
Television time			0.287		0.885			0.248			0.304	0.052
Low (<3 h/day)	130 (20.3)	1.0		1.0		73 (17.8)	1.0		57 (24.9)	1.0		
High (≥3 h/day)	51 (17.3)	0.82 (0.58, 1.18)		1.03 (0.70, 1.51)		19 (10.6)	0.71 (0.40, 1.26)		32 (28.1)	1.33 (0.77, 2.29)		

*GDM* gestational diabetes mellitus, *BMI* body mass index, *OR* odds ratio, *CI* confidence interval

A^a^Adjusted for maternal age, ethnicity, education, pre-pregnancy BMI, parity at recruitment, history of GDM in previous pregnancy, family history of diabetes, dietary energy intake, active/passive smoking during pregnancy and pregnancy weight gain at 26–28 gestational visit

B^b^Adjusted for maternal age, ethnicity, education, parity at recruitment, history of GDM in previous pregnancy, family history of diabetes, dietary energy intake, active/passive smoking during pregnancy and pregnancy weight gain at 26–28 gestational visit

*p*-values were determined by multivariable logistic regression

The findings were substantively similar in terms of magnitude and statistical significance after PA and total sitting time, and PA and television viewing time were mutually adjusted in separate models to examine independent associations of PA, total sitting time and television time with FG and 2hPG levels and GDM (data not shown).

## Discussion

In our study, levels of PA were inversely associated with 2hPG levels and the likelihood of having GDM, but not with FG levels. Compared to insufficiently active women, highly active women had lower 2hPG levels and were less likely to develop GDM. These associations were stronger among overweight/obese women; compared to insufficiently active women, sufficiently and highly active women were less likely to have GDM. Total sitting time was not associated with FG and 2hPG level, or with GDM. Some possible differences between the BMI subgroups were noted, but they were not consistent and require further investigation. Television viewing time was also not associated with glucose levels and GDM.

Our study in a multi-ethnic Asian population generally supports results of a previous meta-analysis reporting inverse associations between PA during pregnancy and risk of developing GDM [6]. Demspsy et al., observed similar results, indicating that women who engaged in any recreational PA during pregnancy experienced a 48% reduction in GDM risk compared with inactive women [49]. There are a limited number of observational studies that examined the association of PA and maternal glucose concentration in the absence of GDM [24, 32]. Chasen-Taber et al. reported a 50% lower risk of abnormal glucose tolerance among women with high levels of moderate-intensity activity as compared with women in the lowest quartile during pregnancy [32]. Higher levels of PA were associated with a lower 2hPG level in our study, which indicates the importance of PA on glucose tolerance in pregnant women; as a consequence it may also have an effect in preventing adverse pregnancy outcome. For instance, two United Kingdom (UK) cohort studies have reported that higher maternal glucose levels within the non-diabetes range were consistently related to adverse pregnancy outcome [51].

The stratified analysis further highlights that these associations were mainly observed in overweight/obese women. Pre-pregnancy BMI may be an effect modifier for the association of level of PA with glucose concentration and GDM. However, this effect was evaluated in only four previous studies [24, 25, 49, 52]. In contrast to our results, the majority of previous studies indicated either no difference in the association between PA and GDM according to maternal BMI [49] or that the association was only found among women with a pre-pregnancy BMI of <25 kg/m^2^ [24]. Deierlein AL et al. reported that women with a pre-pregnancy BMI of <25 kg/m^2^ who engaged in any recreational moderate and vigorous physical activities during pregnancy had a 48% reduced risk of hyperglycemia compared to women who reported no moderate and vigorous activity [52]. The inconsistency between studies might be related to methodological issues, such as study design, population and assessment methods, but also highlights the need for further investigation of these observations. A potential biological mechanism behind the observed associations may be that skeletal muscle contraction as a result of PA may trigger glucose uptake and promote insulin sensitivity [24, 53]. PA-induced reductions in fat mass and increases in muscle mass may also lead to improved glucose tolerance and increased insulin sensitivity. Adipose tissue may also play a significant role in glucose clearance in physically active individuals, especially in obese individuals where this tissue can be considerable enough to contribute to improved glucose metabolism following physical training [49].

In our study, there was no evidence for an association of sedentary behavior with glucose levels and GDM. Nonetheless, we observed consistent increased odds of having GDM in women sitting for ≥7 h/day, but this was not led to statistical significant, which may be due to a lack of statistical power. A previous study that used television viewing time as one of the measures of sedentary behavior also reported no association with abnormal glucose tolerance and GDM risk [24], while another study found that longer sitting time was associated a with higher risk of developing gestational diabetes [30]. These inconsistencies warrant further research.

GUSTO is the first multi-ethnic birth cohort study in Asia investigating the associations of PA, total sitting time and television time during pregnancy with blood glucose levels and GDM. PA and SB data were collected as part of a structured questionnaire, administered by trained interviewers to mitigate the likelihood of systematic reporting errors. Standardized objective methods were used to quantify maternal blood glucose levels, and to determine GDM. Nevertheless, limitations of our study should be considered when interpreting our findings. Firstly, PA and SB data were limited to subjects’ self-reports with potential recall bias due to their complexity, and the questionnaire has not been validated locally against objective methods. Hence, some women might have misclassified their PA intensity categories. Secondly, assigned MET values and categorization of total MET values are suggested for an adult, and not specifically for pregnant women. However, we believe that the magnitude of imprecision is acceptable as the PA assessment was restricted to the first two trimesters of pregnancy. Thirdly, the prevalence of GDM was determined by 1999 WHO diagnostic criteria because only FG and 2hPG data were available, and more recent definitions could not be applied [36]. However, this study also examined the associations of PA and SB with maternal glucose concentrations, which are not influenced by these cut-off criteria for diagnosis. Fourthly, pre-pregnancy BMI was derived from self-reported weight which might have led to misclassification of some women in BMI categories. However, the literature suggests that pre-pregnancy BMI category by self-reported and measured weight are identical for most women [54] Finally, the generalizability of our results is limited because our cohort was not representative of the general Singaporean population [39].

## Conclusions

PA was inversely associated with 2hPG levels and GDM, but not associated with FG levels. Highly active women during pregnancy had significantly lower levels of 2hPG and were less likely to be diagnosed with GDM, especially among those who were overweight/obese. Overweight/obese women with the minimum recommended or higher levels of PA were less likely to have GDM. In contrast, there was little evidence of an association between SB (total sitting time or TV viewing time) and glucose levels in our study. These findings suggest the importance of recommending or maintaining sufficiently high levels of physical activity during pregnancy to reduce the risk of adverse pregnancy outcomes for mothers and their offspring due to hyperglycemia. More prospective longitudinal studies and further trials are warranted, combining subjective and objective assessment of PA and SB with activity specific information, and more precise assessment of GDM, to confirm these findings. This knowledge will help to develop more effective health promotion strategies to prevent hyperglycemia among Asian women.

## Abbreviations

2hPG: 2-h postprandial glucose
BMI: Body mass index
CI: Confidence interval
FG: Fasting glucose
GDM: Gestational diabetes mellitus
GUSTO: Growing Up in Singapore Towards healthy Outcomes
IPAQ: International Physical Activity Questionnaire
KKH: KK Women’s and Children’s Hospital
MET: metabolic equivalent tasks
NUH: National University Hospital
OGTT: Oral Glucose Tolerance Testing
OR: Odds ratio
PA: Physical activity
SB: Sedentary behavior
UK: United Kingdom
WHO: World Health Organization
